# Age‐Specific Associations of Usual Blood Pressure Variability With Cardiovascular Disease and Mortality: 10‐Year Diabetes Mellitus Cohort Study

**DOI:** 10.1161/JAHA.120.019026

**Published:** 2021-08-16

**Authors:** Eric Yuk Fai Wan, Esther Yee Tak Yu, Weng Yee Chin, Jessica K. Barrett, Ian Chi Kei Wong, Esther Wai Yin Chan, Celine Sze Ling Chui, Shiqi Chen, Cindy Lo Kuen Lam

**Affiliations:** ^1^ Department of Family Medicine and Primary Care The University of Hong Kong Hong Kong SAR China; ^2^ Centre for Safe Medication Practice and Research Department of Pharmacology and Pharmacy The University of Hong Kong Hong Kong SAR China; ^3^ Laboratory of Data Discovery for Health (D24H) Hong Kong Science and Technology Park Sha Tin Hong Kong, China; ^4^ Department of Family Medicine and Primary Care The University of Hong Kong Shenzhen Hospital Shenzhen China; ^5^ Medical Research Council (MRC) Biostatistics Unit University of Cambridge Cambridge United Kingdom; ^6^ Research Department of Practice and Policy School of Pharmacy University College London London United Kingdom

**Keywords:** blood pressure, cardiovascular disease, diabetes mellitus, mortality, visit‐to‐visit variability, High Blood Pressure, Risk Factors, Cardiovascular Disease

## Abstract

**Background:**

The detrimental effects of increased variability in systolic blood pressure (SBP) on cardiovascular disease (CVD) and mortality risk in patients with diabetes mellitus remains unclear. This study evaluated age‐specific association of usual SBP visit‐to‐visit variability with CVD and mortality in patients with type 2 diabetes mellitus.

**Methods and Results:**

A retrospective cohort study investigated 155 982 patients with diabetes mellitus aged 45 to 84 years without CVD at baseline (2008–2010). Usual SBP variability was estimated using SBP SD obtained from a mixed‐effects model. Age‐specific associations (45–54, 55–64, 65–74, 75–84 years) between usual SBP variability, CVD, and mortality risk were assessed by Cox regression adjusted for patient characteristics. After a median follow‐up of 9.7 years, 49 816 events (including 34 039 CVD events and 29 211 mortalities) were identified. Elevated SBP variability was independently, positively, and log‐linearly associated with higher CVD and mortality risk among all age groups, with no evidence of any threshold effects. The excess CVD and mortality risk per 5 mm Hg increase in SBP variability within the 45 to 54 age group is >3 times higher than the 70 to 79 age group (hazard ratio, 1.66; 95% CI, 1.49–1.85 versus hazard ratio, 1.19; 95% CI, 1.15–1.23). The significant associations remained consistent among all subgroups. Patients with younger age had a higher association of SBP variability with event outcomes.

**Conclusions:**

The findings suggest that SBP visit‐to‐visit variability was strongly associated with CVD and mortality with no evidence of a threshold effect in a population with diabetes mellitus. As well as controlling overall blood pressure levels, SBP visit‐to‐visit variability should be monitored and evaluated in routine practice, in particular for younger patients.

**Nonstandard Abbreviations and Acronyms**
DM: diabetes mellitus
HA: Hospital Authority
SBP: systolic blood pressure
T2DM: type 2 diabetes mellitus

**Clinical Perspective**

**What Is New?**

- Since visit‐to‐visit variability in systolic blood pressure is a relatively new concept in the clinical field, its effects on cardiovascular disease and mortality risk in patients with diabetes mellitus remain unclear.
- This large cohort study updates our understanding by using a longer follow‐up period and using statistical methods to correct for regression dilution bias to evaluate the age‐specific association between usual systolic blood pressure variability and risk of cardiovascular disease and mortality among Chinese patients with diabetes mellitus.

**What Are the Clinical Implications?**

- As well as controlling overall blood pressure levels, systolic blood pressure visit‐to‐visit variability should be monitored and evaluated in routine practice, in particular for younger patients.

Diabetes mellitus (DM) is one of the most common chronic diseases, affecting 425 million people worldwide, and the number is estimated to rise to 629 million by the year 2045.^1^ With a high prevalence of 8.8%, DM is a source of great burden for global health care because of its high incident complications.^2^ Compared with patients without DM, patients with DM have at a 2‐ to 3‐fold higher risk of cardiovascular disease (CVD), which is a key factor contributing to mortality and disability, leading to 5 million deaths in 2015.^3^ The benefits of blood pressure control, in particular systolic blood pressure (SBP), in preventing CVD is well studied in populations with DM,^4^, ^5^, ^6^, ^7^ and thus several international guidelines on DM management have established the SBP target.^8^, ^9^ Since visit‐to‐visit variability in SBP is a relatively new concept in the clinical field and more solid evidence is needed to prove its value in risk prediction. The effect of SBP variability has received particular attention in the context of patients with DM.

A recent systematic review found a relationship between long‐term SBP variability and higher risk of CVD and mortality in the population with DM.^10^ However, only 7 of 18 studies included in the review contributed results for the effect of the long‐term variability of SBP on CVD and mortality outcomes (6 for CVD; 5 for mortality). One of the more recent studies included in this review was conducted in a Swedish population with DM managed in primary care settings. It showed that SBP variability was associated with all‐cause mortality only for patients without CVD and not taking blood pressure (BP)‐lowering medications but not for other patients with DM.^11^ Furthermore, among these 7 studies, 4 studies had a small (n<3000) number of patients, and 5 studies had <10 years of follow‐up. More importantly, the measures of SBP variability are estimated on a person‐by‐person basis and thus subject to measurement error, which causes regression dilution bias in the estimated association between SBP variability and event outcomes.^12^ In addition, the association between SBP and cardiovascular diseases may vary in different age groups,^13^, ^14^, ^15^ but there is no available study evaluating the age‐specific effects of usual SBP variability on CVD and mortality among patients with DM.

This cohort study updates our previous study using a longer follow‐up period (10 years versus 5 years) and using statistical methods to correct for regression dilution bias to evaluate the age‐specific association between usual SBP variability and CVD and mortality in patients with type 2 DM (T2DM) managed in Hong Kong primary care settings.^16^ The pattern of variability among patients with different baseline characteristics was also explored. More comprehensive knowledge of the effect of usual SBP variability could help enable clinicians to better identify patients at higher risk earlier on.

## METHODS

Because of the confidentiality of the data used for this study and strict privacy policy from the data holder that the data can be kept among the designated research personnel only, the data cannot be provided to anyone else, irrespective of whether or not the data are anonymized.

### Study Design

This is a retrospective cohort study, which obtained the data from the prospectively collected population clinical database from the Hong Kong Hospital Authority (HA). Patients were included if they were aged 45 to 84 years, with a clinical diagnosis of T2DM, with no prior diagnosis of CVD at baseline, and managed in public primary care clinics of the Hong Kong HA from January 1, 2008, to December 31, 2010. The HA is the chief regulatory institution for all 42 public‐sector hospitals, 47 specialist outpatient clinics, and 73 primary care clinics in Hong Kong. Over 90% of the local population with chronic disease receive medical treatments through the HA.^17^ No consent was needed from the subjects because the data were analyzed anonymously. Diagnosis of T2DM was made by HA clinic doctors and recorded using the International Classification of Primary Care‐2 code of T90. All confidential data, including patients' characteristics and event outcomes, are kept in the electronic health database of the HA's clinical management system. The validity and coding accuracy of the clinical management system database has been well established and adopted in several previous high‐quality population‐based epidemiologic studies.^16^, ^18^, ^19^ Clinicians and other healthcare professionals receive training in using the clinical management system to record a variety of the clinical information and patient demographics simultaneously during the doctor's consultation, including patients' diagnoses, prescriptions, laboratory tests and results, emergency department visits, hospitalizations, and specialist and primary care outpatient clinic visits. The timeline of the measurements of SBP and outcome determination is presented in Figure S1. Most patients with DM managed in public primary care clinics are typically followed‐up once per quarter. Hence, a first SBP reading with a repeated reading every 3 months until the end of the 2‐year follow‐up (total 9 readings) was used to estimate the SBP variability. Each patient was continuously tracked until the incident date of the outcome event, death, or the last follow‐up visit before December 31, 2017, whichever occurred first.

### Outcome Measures

The primary outcome was the incidence of the composite of CVD and all‐cause mortality. The secondary outcomes consisted of individual CVD, the subtype of coronary heart disease (CHD), stroke, heart failure, all‐cause mortality, CVD mortality, and non‐CVD mortality. The outcome events were defined using the International Classification of Primary Care‐2 or the *International Classification of Diseases, Ninth Edition, Clinical Modification* (*ICD‐9‐CM*), which are officially recognized diagnostic codes. The Hong Kong Government Death Registry provided the mortality reports retrieved from their internal population data, where CVD‐related mortality was classified as death with a history of CVD or the main cause of death was recorded according to the *International Classification of Diseases, Tenth Edition* codes of I20 to I25, I50, and I60 to I69. In a previous local study, these codes have revealed a high coding accuracy in diagnosing myocardial infarction and stroke with positive predictive values of 85.4% (95% CI, 78.8%–90.6%) and 91.1% (83.2%–96.1%), respectively.^19^ Table S1 also summarizes all event definitions.

### Ethical Approval

Institutional review boards of the Hong Kong Hospital Authority have reviewed and approved the ethical examination in this study.

### Clinical BP Measurements

In the process of obtaining and documenting SBP readings in patients during the consultations, all clinics strictly followed a standardized guideline.^20^ Patients are instructed to maintain a seated position for 5 minutes without any distraction before the start of measurements of SBP, and to have at least 1 minute of rest between 2 measurements of SBP. Nurses or trained patient care assistants used a standardized automated sphygmomanometer (UA‐853, Tokyo, Japan; EDAN M3A, Shenzhen, P.R. China; or other equivalent measurements). Previous studies showed that the technology used by the UA‐853 has been clinically validated by the general population.^21^ However, a validation study for EDAN M3A or other equivalent measurements is unavailable. If the difference between the 2 readings exceeded 5 mm Hg, an extra measurement would be taken. The average of all SBP readings taken at a single visit was calculated and recorded as the SBP measurement result for the corresponding visit in the database. Note that, given the limitation of the setting, the BP measurements were not necessarily implemented at the same time of day for each patient.

### Usual SBP and SBP Variability Measurements

To minimize measurement error, and therefore regression dilution bias in the corresponding hazard ratio, usual SBP, and SBP variability measures were obtained using a mixed effects model, which allows the within‐individual variability to differ between individuals. (Regression dilution bias occurs when random measurement error in the values of an explanatory variable causes an attenuation or “flattening” of the slope of the regression line.) Longitudinal trajectories were modeled by including a slope term as both a fixed and random effect. Bayesian Markov chain Monte Carlo was used to fit the mixed‐effects model. The usual SBP and SBP variability were estimated by the posterior mean of the random intercept and the residual standard deviation, respectively. The mixed‐effects model was not adjusted for other covariates. In general, the usual SBP and SBP variability were the individual (subject‐specific) mean and SD of SBP from the mixed‐effects model. Details of the calculation of the usual SBP and SBP variability measures have been described in Data S1 and elsewhere in the literature.^12^ Patients with <4 SBP records were excluded from this study to increase the precision of the usual SBP and SBP variability estimates. The average total number of SBP measurements was 4.5 (SD, 1.3; range, 4–9). The analysis was implemented using JAGS Version 3.4.0 and the R2jags package in R.^22^, ^23^

### Baseline Characteristics

Baseline covariates encompassed sex, age, smoking status, body mass index (BMI), SBP, diastolic blood pressure, hemoglobin A1c, low‐density lipoprotein cholesterol, estimated glomerular filtration rate (eGFR), the Charlson comorbidity index (a composite index representing a patient's comorbid conditions),^24^, ^25^ the use of anti‐hypertensive drugs (eg, angiotensin‐converting enzyme inhibitor or angiotensin receptor blocker, β‐blocker, calcium channel blocker, diuretics, and others [hydralazine, methyldopa, and prazosin]), oral antidiabetic drugs, insulin, and lipid‐lowering agents. The latest record on or before baseline (defined in Figure S1) was used. The eGFR was computed in accordance with the creatinine level from blood testing along with the abbreviated Modification of Diet in Renal Disease Study formula recalibrated for Chinese (eGFR in mL/min per 1.73 m^2^=186×[(serum creatinine in μmol/L)×0.011]−1.154×(age)^−0.203^×(0.742 if female)×1.233), where 1.233 is the adjusted coefficient for local population.^26^ All laboratory assays were performed in accredited laboratories by the College of American Pathologists, the Hong Kong Accreditation Service, or the National Association of Testing Authorities, Australia.

### Statistical Analysis

Multiple imputation was used to impute missing data for all baseline characteristics apart from blood pressure. The chained equation method was used to impute each missing value 5 times, adjusted for all baseline covariates and outcomes including CVD and all‐cause mortality. The same analysis method was adopted for each of the 5 imputed data sets, and the results were pooled using Rubin's rule.^27^

All patients were allocated into 1 of 5 groups defined by their usual SBP variability (<10, 10–11.9, 12–13.9, 14–15.9, and ≥16 mm Hg). After multiple imputation, descriptive statistics were displayed for integration of the baseline characteristics for each group. The cumulative incidence and incidence rate of CVD, mortality, and their composite events were reported, and the CI of the incidence rate was estimated using the Poisson distribution. Age‐specific associations (45–54, 55–64, 65–74, and 75–84 years) between usual SBP variability and the risk of an event were estimated by multivariable Cox proportional hazards regressions adjusted for usual SBP and all baseline covariates including the use of antihypertensive drugs. The hazard ratio (HR), which is a measure of the effect of SBP variability on the hazard of the event of interest, and its CI were reported. An additional term was fitted to allow the HR within each age group to be assessed as the geometric mean of the HRs in the first and second half of that decade.^28^ Thus, the HRs comparing event risk between different SBP variability groups were not assumed to be similar for different age decades. The 95% CIs of the HRs were estimated using the floating absolute risk, without the requirement of selecting a reference group for displaying the standard error.^29^ Details of the above methods have previously been described in the literature,^28^, ^29^ and they have been widely adopted in several large epidemiologic studies.^30^, ^31^, ^32^ To confirm the shape of the association, Cox models were fitted with SBP variability treated as a continuous variable and using restricted cubic splines with 3 knots to model the association.^33^ To enhance the robustness of the results and minimize the potential bias attributable to multiple imputation, reverse causality, and the number of SBP measurements, 5 sensitivity analyses were conducted. First, Rosner's regression method, rather than the mixed‐effects model, was used to adjust for regression dilution bias.^34^ The details of this approach are described in Data S1. Second, a complete case analysis was performed. Third, patients were excluded if they attended <1‐year of follow‐up. Fourth, the analyses were implemented for patients with ≥2 and ≥7 rather than ≥4 SBP measurements to evaluate the potential selection bias attributable to the cutoff in the number of SBP measurements. Fifth, the use of aspirin was included as one of the covariates in the regression analysis.

To further explore the effect of usual SBP variability on the outcomes for patients with different characteristics, subgroup analyses were performed, which were stratified by sex (male, female), age at risk (45–54, 55–64, 65–74, 75–84 years), smoking status (nonsmoker, smoker), BMI (<25, ≥25 kg/m^2^), baseline SBP (<130, 130–139, 140–149, ≥150 mm Hg), usual SBP (<130, 130–139, 140–150, ≥150 mm Hg), low‐density lipoprotein cholesterol (<2.6, ≥2.6 mmol/L), hemoglobin A_1c_ (<7, ≥7%), eGFR (<90, ≥90 mL/min per 1.73 m^2^), Charlson index (<4, ≥4), the number of types of antihypertensive drugs prescribed (0, 1, 2, 3), and the use of different antihypertensive drugs.

All significance tests were 2‐tailed and those with a *P*<0.05 were considered statistically significant. The statistical analysis was executed in Stata Version 13.0.

## RESULTS

### Patient Population

A cohort of 155 982 patients with T2DM satisfied the inclusion criteria and was included in the analyses. Table S2 illustrates that the data completion rate for all baseline characteristics was higher than 84.3%, except for eGFR (77.1%), BMI (64.6%), and low‐density lipoprotein cholesterol (63.6%). In Table 1, patient characteristics are summarized by usual SBP variability group at baseline after multiple imputation. Overall, the mean age was 63.9 years (SD, 10.0) and men account for 45.2%. Usual SBP mean and SBP variability were 137.1 mm Hg (SD, 10.7) and 12.7 mm Hg (SD, 2.3), respectively.

**Table 1:** Descriptive Statistics for Baseline Characteristics Among Patients Stratified by SBP Variability

	SBP variability					Overall (N=155 982)
	<10 mm Hg (N=7315)	10–11 mm Hg (N=42 878)	12–13 mm Hg (N=59 851)	14–15 mm Hg (N=29 422)	≥16 mm Hg (N=16 516)	
Baseline characteristics						
Male, %	47.6	47.6	44.9	44.1	41.4	45.2
Age, y	58.3±9.2	61.6±9.8	64.2±9.8	65.9±9.7	67.2±9.7	63.9±10.0
Current smoker	13.6	11.4	9.8	9.5	9.3	10.3
SBP, mm Hg	114.3±9.3	128.4±11.1	138.4±13.8	143.7±17.9	149.6±25.2	136.7±17.6
DBP, mm Hg	69.2±8.1	74.0±9.2	75.9±9.8	76.4±10.8	77.2±12.4	75.3±10.2
HbA_1c_, %	7.3±1.5	7.4±1.4	7.4±1.5	7.4±1.5	7.4±1.5	7.4±1.4
BMI, kg/m^2^	24.6±4.4	25.4±4.4	25.7±4.5	25.7±5.5	25.8±4.8	25.6±4.9
LDL‐C, mmol/L	3.1±0.9	3.1±1.0	3.2±1.0	3.2±1.0	3.2±1.0	3.2±1.1
eGFR, mL/min per 1.73 m^2^	109.3±33.4	104.0±41.6	99.8±73.9	96.3±47.4	92.5±46.8	100.0±62.1
Charlson index	2.4±1.1	2.7±1.2	3.0±1.2	3.2±1.3	3.4±1.3	3.0±1.3
Use of oral antidiabetic drugs	79.7	80.5	81.5	81.0	80.9	81.0
Use of insulin	1.1	0.7	0.9	1.2	1.7	1.0
Use of ACEI/ARB	11.1	20.4	31.9	39.2	44.6	30.5
Use of β‐blocker	16.8	23.5	29.2	34.9	44.6	29.8
Use of CCB	17.3	29.0	37.7	47.4	59.2	38.4
Use of diuretic	7.9	12.3	14.4	15.1	15.9	13.8
Use of other antihypertensive drugs	5.1	7.6	10.8	13.5	17.1	10.8
Use of lipid‐lowering agents	7.9	9.8	9.8	10.0	10.6	9.8
Variables assessed during the assessment period						
Number of SBP measurements	4.7±1.5	4.6±0.6	4.5±0.4	4.5±0.6	4.6±0.9	4.5±1.3
Usual SBP, mm Hg	116.8±6.9	129.6±3.0	138.5±2.5	143.3±3.8	149.2±5.8	137.1±10.7
SBP variability, mm Hg	9.0±0.4	10.7±0.2	12.5±0.2	14.3±0.3	17.4±1.2	12.7±2.3

All parameters are expressed in either percentage or mean (SD). Other antihypertensive drugs included acarbose, glucagon‐like peptide‐1 agonist, meglitinides. ACEI indicates angiotensin‐converting enzyme inhibitor; ARB, angiotensin receptor blocker; BMI, body mass index; CCB, calcium channel blocker; DBP, diastolic blood pressure; eGFR, estimated glomerular filtration rate; HbA_1c_, hemoglobin A_1c_; LDL‐C, low‐density lipoprotein cholesterol; and SBP, systolic blood pressure.

### Outcomes

**Figure 1:**
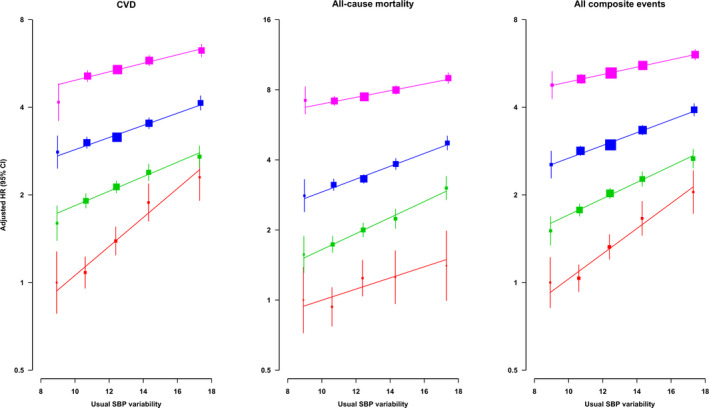
Age‐specific adjusted HRs for the risk of CVD, all‐cause mortality, and their composite with increasing usual SBP variability by multivariable Cox regressions. HR was adjusted by age at risk, sex, smoking status, body mass index, SBP, diastolic blood pressure, hemoglobin A_1c_, low‐density lipoprotein cholesterol, estimated glomerular filtration rate, the usages of oral antidiabetic drugs, insulin, angiotensin converting enzyme inhibitor/angiotensin receptor blocker, β‐blocker, calcium channel blocker, diuretic, other antihypertensive drugs, lipid‐lowering agent, Charlson's index, and usual SBP. Red, green, blue, and pink line were 45 to 54, 55 to 64, 65 to 74, and 75 to 84 age at‐risk group. The area of each square was inversely proportional to the variance of the category‐specific log risk. CIs are displayed as floating absolute risks. CVD indicates cardiovascular disease; HR, hazard ratio; and SBP, systolic blood pressure.

Table 2 demonstrates the cumulative incidence and incidence rate of CVD, all‐cause mortality, and their composite event by usual SBP variability group. After a median follow‐up of 9.7 years (16.4 million person‐years), the overall number of any event is 49 816 incidents, incorporating 34 039 CVDs and 29 211 mortalities. There was a trend for both cumulative incidence and incidence rates of all outcomes to increase with SBP variability, from the lowest group (usual SBP variability <10 mm Hg) to the highest group (usual SBP variability ≥18 mm Hg), including CVD from 12.8 to 42.7, mortality from 10.3 to 34.2, and all events from 20.6 to 60.9 per 1000 person‐years. Age‐specific associations are shown in Figure 1 and Figure S2. Figure S3 presents the positive and log‐linear association (curvilinear) between usual SBP variability and all outcomes after adjustment for the patient's baseline characteristics. The results of the Cox models with a restricted cubic spline SBP variability association also suggested direct log‐linear associations, as indicated in Figure S4. In general, positive and log‐linear associations are found between SBP and the risk of CVD, including CHD, stroke, heart failure, all‐cause mortality, CVD mortality, non‐CVD mortality, and their composite event for all age groups.

**Table 2:** Number, Incidence Rate, and Hazard Ratio of CVD and Mortality, Stratified by SBP Variability

	SBP variability				
	<10 mm Hg (N=7315)	10–12 mm Hg (N=42878)	13–15 mm Hg (N=59 851)	16–18 mm Hg (N=29 422)	≥18 mm Hg (N=16 516)
CVD					
Cumulative cases with event	819	7306	13 031	7731	5152
Incidence rate (95% CI)*	12.8 (12.0–13.8)	20.1 (19.6–20.6)	26.6 (26.1–27.0)	33.7 (32.9–34.4)	42.7 (41.5–43.9)
Hazard ratio^†^ (95% CI)	1.00 (0.92–1.08)	1.18 (1.15–1.21)	1.27 (1.25–1.29)	1.41 (1.37–1.44)	1.58 (1.52–1.64)
Coronary heart disease					
Cumulative cases with event	417	3543	6093	3513	2331
Incidence rate (95% CI)*	6.4 (5.8–7.0)	9.4 (9.1–9.7)	11.8 (11.5–12.1)	14.3 (13.8–14.7)	17.7 (17.0–18.5)
Hazard ratio^†^ (95% CI)	1.00 (0.90–1.12)	1.15 (1.11–1.20)	1.19 (1.17–1.22)	1.27 (1.23–1.33)	1.42 (1.35–1.50)
Stroke					
Cumulative cases with event	381	3614	6500	3990	2667
Incidence rate (95% CI)*	5.8 (5.3–6.4)	9.6 (9.2–9.9)	12.6 (12.3–12.9)	16.3 (15.8–16.8)	20.5 (19.8–21.3)
Hazard ratio^†^ (95% CI)	1.00 (0.89–1.12)	1.26 (1.21–1.31)	1.39 (1.36–1.42)	1.59 (1.53–1.65)	1.81 (1.72–1.90)
Heart failure					
Cumulative cases with event	126	1693	3726	2406	1821
Incidence rate (95% CI)*	1.9 (1.6–2.3)	4.4 (4.2–4.6)	7.0 (6.8–7.3)	9.5 (9.2–9.9)	13.6 (12.9–14.2)
Hazard ratio^†^ (95% CI)	1.00 (0.82–1.23)	1.38 (1.30–1.46)	1.48 (1.44–1.53)	1.56 (1.49–1.64)	1.76 (1.65–1.87)
All‐cause mortality					
Cumulative cases with event	689	5976	11 070	6707	4769
Incidence rate (95% CI)*	10.3 (9.6–11.1)	15.3 (14.9–15.6)	20.5 (20.1–20.9)	25.9 (25.3–26.5)	34.2 (33.3–35.2)
Hazard ratio^†^ (95% CI)	1.00 (0.91–1.09)	1.06 (1.03–1.10)	1.14 (1.12–1.16)	1.25 (1.21–1.29)	1.46 (1.40–1.52)
Cardiovascular mortality					
Cumulative cases with event	209	2501	5186	3441	2670
Incidence rate (95% CI)*	3.1 (2.7–3.6)	6.4 (6.1–6.6)	9.6 (9.3–9.9)	13.3 (12.9–13.8)	19.2 (18.5–19.9)
Hazard ratio^†^ (95% CI)	1.00 (0.85–1.18)	1.26 (1.20–1.33)	1.38 (1.34–1.42)	1.59 (1.52–1.66)	1.90 (1.79–2.01)
Non‐cardiovascular mortality					
Cumulative cases with event	480	3475	5884	3266	2099
Incidence rate (95% CI)*	7.2 (6.6–7.8)	8.9 (8.6–9.2)	10.9 (10.6–11.2)	12.6 (12.2–13.1)	15.1 (14.5–15.7)
Hazard ratio^†^ (95% CI)	1.00 (0.90–1.11)	1.02 (0.97–1.06)	1.08 (1.06–1.11)	1.13 (1.09–1.18)	1.26 (1.19–1.34)
All composite events					
Cumulative cases with event	1311	10 893	19 129	11 138	7345
Incidence rate (95% CI)*	20.6 (19.5–21.7)	30.0 (29.4–30.5)	39.0 (38.4–39.5)	48.5 (47.6–49.4)	60.9 (59.5–62.3)
Hazard ratio^†^ (95% CI)	1.00 (0.94–1.07)	1.13 (1.10–1.15)	1.22 (1.20–1.23)	1.33 (1.30–1.36)	1.50 (1.46–1.55)

CKD indicates chronic kidney disease; CVD, cardiovascular disease; and SBP, systolic blood pressure.

* Incidence rate (cases/1000 person‐years) with 95% CI based on Poisson Distribution.

^†^ Hazard ratio was adjusted by age at risk, sex, smoking status, body mass index, SBP, diastolic blood pressure, hemoglobin A_1c_, low‐density lipoprotein‐cholesterol, estimated glomerular filtration rate, the usages of oral antidiabetic drugs, insulin, angiotensin‐converting enzyme inhibitor/angiotensin receptor blocker, β‐blocker, calcium channel blocker, diuretic, other antihypertensive drugs, lipid‐lowering agent, Charlson's index and usual SBP.

**Figure 2:**
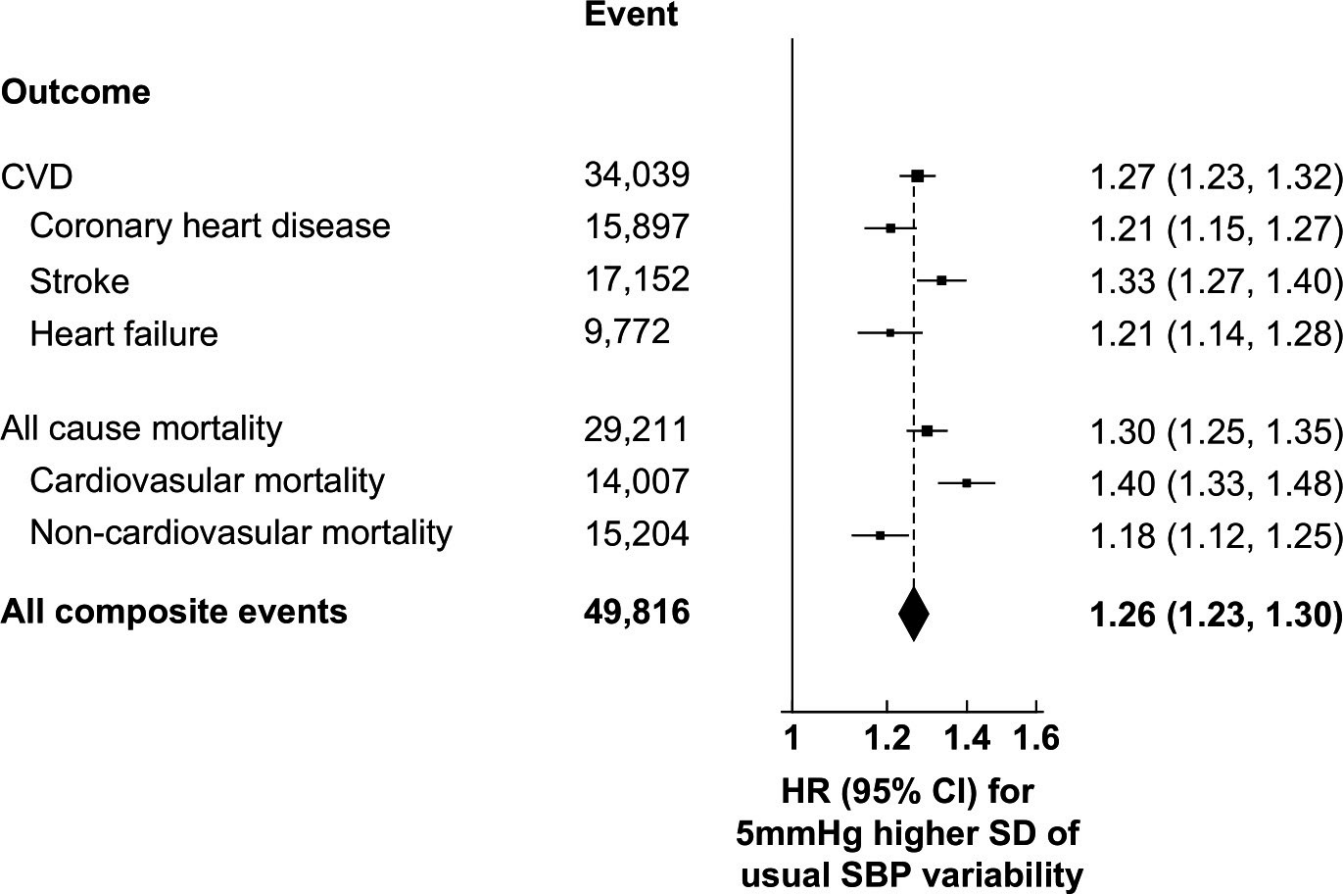
Adjusted HRs for the risk of CVD, coronary heart disease, stroke, heart failure, all‐cause mortality, CVD mortality, non‐CVD mortality, and their composite with each 5 mm Hg increasing usual SBP variability using multivariable Cox regression. HR was adjusted by age at risk, sex, smoking status, body mass index, SBP, diastolic blood pressure, hemoglobin A_1c_, low‐density lipoprotein cholesterol, estimated glomerular filtration rate, the usages of oral antidiabetic drugs, insulin, angiotensin‐converting enzyme inhibitor/angiotensin receptor blocker, β‐blocker, calcium channel blocker, diuretic, other antihypertensive drugs, lipid‐lowering agent, Charlson's index, and usual SBP. Squares show the HR while the diamond shows the pooled HR, and the dotted line indicates the center of the diamond. CVD indicates cardiovascular disease; HR, hazard ratio; and SBP, systolic blood pressure.

Figure 2 demonstrates the relationship between usual SBP variability as a continuous variable and the risk of all outcome events. Each 5 mm Hg increase in usual SBP variability was associated with 27% (HR, 1.27; 95% CI, 1.23–1.32), 30% (HR, 1.30; 95% CI, 1.25–1.35), and 26% (HR, 1.26; 95% CI, 1.23–1.30) higher risk of CVD, all‐cause mortality, and their composite, respectively. Moreover, the HR for stroke (HR, 1.33; 95% CI, 1.27–1.40) is higher than that for CHD (HR, 1.21; 95% CI, 1.15–1.27) and heart failure (HR, 1.21; 95% CI, 1.14–1.28), indicating that the association with CVD may be driven predominantly by stroke. Similarly, the HR for non‐CVD related mortality (HR, 1.18; 95% CI, 1.12–1.25) was lower than that for CVD‐related mortality (HR, 1.40; 95% CI, 1.33–1.48), implying that mortality may be mainly attributable to CVD‐related mortality. Five sensitivity analyses including (1) the use of Rosner's regression method (the regression dilution ratio was 4.13) in Figure S5, (2) complete case analysis in Figure S6, (3) excluding patients with <1‐year follow‐up period in Figure S7, (4) including patients with ≥2 and ≥7 rather than ≥4 SBP measurements in Figures S8 and S9, (5) including the use of aspirin on or before baseline in Figure S10, demonstrated similar results.

### Subgroup Analyses

**Figure 3:**
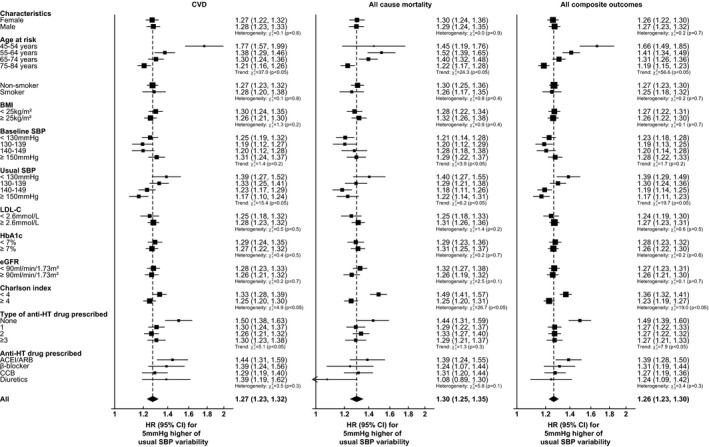
Adjusted HRs for the risk of CVD, all‐cause mortality, and their composite events with each 5 mm Hg increasing usual SBP variability by stratifying patient's characteristics at baseline using multivariable Cox regression. HR was adjusted by age at risk, sex, smoking status, BMI, SBP, diastolic blood pressure, HbA_1c_, LDL‐C, estimated glomerular filtration rate, the usages of oral antidiabetic drugs, insulin, ACEI/ARB, β‐blocker, CCB, diuretic, other antihypertensive drugs, lipid‐lowering agent, Charlson's index, and usual SBP. Squares show the HR while the diamond shows the pooled HR, and the dotted line indicates the centre of the diamond. ACEI indicates angiotensin‐converting enzyme inhibitor; ARB, angiotensin receptor blocker; BMI, body mass index; CCB, calcium channel blocker; CVD, cardiovascular disease; eGFR, estimated glomerular filtration rate; HbA_1c_, hemoglobin A_1c_; HR, hazard ratio; HT, hypertensive; LDL‐C, low‐density lipoprotein‐cholesterol; and SBP, systolic blood pressure.

The forest plots in Figure 3 and Figures S11 and S12 show that usual SBP variability is associated with a significant increase in the risk of each outcome event across all selected subgroups. In general, the HRs for the composite of CVD and all‐cause mortality demonstrate that age and usual SBP is inversely related to the SBP variability HR. The HRs are higher among patients with low comorbidity (Charlson index <4), and patients without prescription of antihypertensive drugs. Comparable effects are observed when stratified by male patients, smoking status, BMI, baseline SBP, hemoglobin A_1c_, eGFR, and low‐density lipoprotein cholesterol, and use of diverse antihypertensive drugs. The age at which at‐risk subgroups have the most pronounced difference in SBP variability HRs for the risk of CVD and all‐cause mortality, with a 66% higher risk per 5 mm Hg increase is in the 45 to 54 age group (all composite outcomes HR, 1.66; 95% CI, 1.49–1.85), compared with a 19% higher risk in the 70 to 79 age group (all composite outcomes HR, 1.19; 95% CI, 1.15–1.23), which indicates that the excess risk in the 45 to 54 age group per 5 mm Hg increase in SBP variability is >3 times higher than for the 70 to 79 age group. A similar trend for the usual SBP was obtained in that patients with usual SBP <130 mm Hg had doubled the risk compared with those with usual SBP ≥150 mm Hg (HR, 1.39; 95% CI, 1.29–1.49 versus 1.17; 95% CI, 1.11–1.23). Different effects of usual SBP variability were observed in terms of the comorbidity (HR, 1.36; 95% CI, 1.32–1.41] in Charlson index <4 versus 1.23; 95% CI, 1.19–1.27 in Charlson index ≥4) and the number of types of antihypertensive drug prescribed (HR, 1.49; 95% CI, 1.39–1.60 in none versus 1.27; 1.21–1.33 in ≥3 types).

## Discussion

This large cohort study is the first to estimate usual SBP variability using mixed‐effects models to reduce regression dilution in the estimated association with outcome events, and to determine the positive and log‐linear age‐specific association between usual SBP variability and risk of CVD, CHD, stroke, heart failure, all‐cause mortality, CVD mortality, and non‐CVD mortality among Chinese patients with DM. The effect of SBP variability on the risk of various outcome events remained significant regardless of patient characteristics after adjusting for usual and baseline SBP, suggesting that SBP variability may provide additional valuable information as a potential predictor for the incidence of CVD events and mortality in the population with DM, irrespective of the absolute SBP readings. There was no threshold for SBP variability, indicating that the lower the SBP variability, the better the outcome. These findings suggest that clinicians should be mindful of the effect of BP fluctuation on the incidence of CVD and all‐cause mortality, as well as the focus on an optimal BP target. Moreover, the impact of usual SBP variability is strengthened in patients at a younger age, lower usual SBP, Charlson index, and the number of types of antihypertensive drugs prescribed. This is suggestive that additional attention may be needed for these specific patient groups when they also experience higher SBP variability.

There is currently no literature investigating the age‐specific effects of usual SBP variability on CVD and mortality among patients with DM. In general, our results demonstrated a direct log‐linear or linear association between SBP variability and outcome events, which confirmed the results of previous studies. For instance, a posttrial follow‐up study of the landmark ADVANCE (Action in Diabetes and Vascular Disease: Preterax and Diamicron Modified Release Controlled Evaluation) trial, on 9113 patients with DM with a medium follow‐up of 7.6 years also illustrated the positive log‐linear associations that each 5 mm Hg increment in SD of SBP was associated with a 13%, 8%, and 11% increase in the risk of all‐cause mortality, CVD, and myocardial infarction, respectively.^35^ No significant association for stroke and CVD mortality was obtained.^35^ Nevertheless, they acknowledged that the small sample size and number of events (668 stroke events and 614 CVD mortality events) may have affected the generalizability of these results. A few studies have shown no evidence of any significant associations between SBP variability and outcome events.^11^, ^36^ In a cohort study on 2161 patients with DM in Taiwan, over a 5.5‐year follow‐up, SBP variability was significantly associated with all‐cause mortality but not with CVD mortality. This was likely attributable to the small sample size and the small number of events as only 25 CVD mortality events among 2161 patients were observed.^36^ Furthermore, the measurement of SBP variability was determined by SBP readings taken after baseline, which may have resulted in informative censoring or immortal time bias.^15^, ^37^ Compared with these studies, we have incorporated a significantly larger number of patients without CVD, longer follow‐up periods, and a much larger number of incident outcome events. In this current study, SBP variability was based on measurements taken before baseline; hence, the results should be less subject to bias and better powered to demonstrate the etiological associations between SBP variability and adverse outcomes. Note that none of the previous studies corrected their results for regression dilution bias, and thus their findings may underestimate the effect of SBP variability. Hence, it is expected that the strength of the association of SBP variability on the risk of event outcomes in this study will be greater.

In this current study, the analyses of individual CVD events identified a stronger impact of SBP variability on stroke compared with CHD and heart failure. Indeed, a recent meta‐analysis, which mostly included either studies involving patients with severe diseases such as CVD or studies of the general population, also showed a similar pattern that SBP variability, as with usual BP,^30^ contributes more to stroke than cardiac events.^15^ Our results extend these findings that the effect of SBP variability on CVD events may be driven primarily by stroke in the population with DM.

A recent post hoc analysis of the VALUE (Valsartan Antihypertensive Long‐Term Use Evaluation) trial on 13 803 patients with hypertension over a mean follow‐up period of 4.2 years reported that SBP variability was associated with a significantly higher risk of multiple diseases in patients aged <68 years than in those aged ≥68 years.^38^ Additionally, our findings from subgroup analyses showed a trend for those within the study age range of 45 to 85 years that not only younger people, but also those with lower‐than‐usual SBP, lower Charlson index, and with fewer types of antihypertensive drugs prescribed, were more susceptible to the adverse influence of SBP variability. The reasons may be that these patients were more likely to have lower SBP variability, as shown in Table 1 and the literature.^39^ As a consequence, these patients may be more vulnerable to differences in SBP variability. Also, patients with these characteristics (eg younger and low comorbidity) were more likely to have fewer vascular risk factors, and thus may be more sensitive to blood pressure variability. In contrast, other risk factors in patients with higher age and morbidity may overshadow the detrimental effect of SBP variability. A few studies have concluded that poor antihypertensive medication adherence and use of different types of antihypertensive drugs may cause patients to be more prone to higher SBP variability and CVD risk.^40^, ^41^, ^42^ However, although drug adherence information was unavailable in this current study, many previous studies have found no significant difference in SBP variability among different levels of drug adherence or types of antihypertensive medications used.^43^, ^44^, ^45^ Therefore, these factors may not explain the links between SBP variability and the incidence of CVD and all‐cause mortality.^39^, ^44^ Further studies are required to clarify the mechanism of this relationship.

### Strengths and Limitations

Our study has several strengths. First, we included a T2DM cohort with nearly 10 years of follow‐up, which to our knowledge was the largest and longest study conducted to date and well powered to demonstrate the associations between usual SBP variability and outcome events in different subgroups. Second, we used appropriate statistical analysis methods to correct for regression dilution bias and conducted sensitivity analyses, which allowed us to make a comprehensive evaluation of the relationship between SBP and diverse clinical outcomes. Multiple imputation was used to impute missing data to reduce selection bias. Third, relevant baseline covariates, such as patients' laboratory results, disease attributes, and treatment modalities, were considered to generate reliable results with the aid of HA's computerized administrative database.

There were also limitations to our study. First, the study design of a retrospective cohort study can yield a conclusion only about association but not causation. However, a low probability of reverse causation was observed as patients with CVD at baseline were excluded in this study, and the results were very similar in the sensitivity analysis when we included only patients with a follow‐up period of over 1 year. Second, potential confounding factors related to lifestyles, such as physical activity level and dietary intake, were not assessed in this study. Instead, we have examined the individuals' disease history and anthropometric and clinical parameters, including BMI, hemoglobin A_1c_, and lipid profile, which could reflect both the severity of their comorbidities and their lifestyle habits. Third, the validation study for the standardized automated sphygmomanometer (EDAN M3A, Shenzhen, China, or other equivalent measurements) used, and the record of adherence to the protocol for measuring BP are not available, and thus the recorded BP measurements may be subject to measurement error. Moreover, covariates such as medication and smoking habits were not included in the calculation of SBP variability. Finally, the association between SBP variability and increased CVD risk was well demonstrated in this study. However, this could be affected by differences between our study population and the general population and the T2DM populations from other Chinese regions. Temporal variations and variations in risk factors or interventions not included in this study might also induce heterogeneity in the association. Therefore, our findings might not be generalizable to other settings.

## Conclusions

In this population‐based cohort study of Chinese primary care patients with T2DM, usual SBP variability was associated with increased CVD risk and all‐cause mortality independently of usual and baseline SBP. We demonstrated positive log‐linear age‐specific associations between usual SBP variability and the risk of CVD, CHD, stroke, heart failure, all‐cause mortality, CVD mortality, and non‐CVD mortality. The clinical implication of these findings is that SBP variability should be monitored and evaluated in routine practice, as this appears to be a risk factor for developing CVD and all‐cause mortality. The impact of usual SBP variability was more significant in patients with younger age, lower‐than‐usual SBP, fewer comorbidities, and fewer types of antihypertensive drugs being prescribed. We suggest that additional monitoring may be needed for these patients if they have higher SBP variability. Future studies are needed to confirm these findings, standardize the definition of the variability of BP, evaluate the prediction power and relative importance between different features of BP (eg, variability and absolute value) and other risk factors, the mechanism of BP variability and CVD, potential interventions for high BP variability, and to determine a therapeutic target for SBP variability to provide better outcomes for patients with T2DM.

## Sources of Funding

This work was supported by the Health Services Research Fund, Food and Health Bureau, HKSAR (Ref. no 14151181). Barrett was funded by the MRC Unit Programme number MC_UU_00002/5. No funding organization had any role in the design and conduct of the study; collection, management, analysis, and interpretation of the data; and preparation of the manuscript.

## Disclosures

Professor Wong received funding from Pfizer, Bayer, and Novartis to evaluate real‐world evidence on pharmacologic treatments of cardiovascular diseases but not related to the current study. Dr Chan received research grants from Bayer, Bristol‐Myers Squibb, Janssen, a Division of Johnson and Johnson, Pfizer, and Takeda to evaluate real‐world evidence on pharmacologic treatments of cardiovascular diseases but not related to the current study. The remaining authors have no disclosures to report.

## Supporting information

Data S1
Tables S1–S2
Figures S1–S12
