# Supplementary material for: Age‐Specific Associations of Usual Blood Pressure Variability With Cardiovascular Disease and Mortality: 10‐Year Diabetes Mellitus Cohort Study
Source: J Am Heart Assoc. 2021 Aug 16;10(17):e019026. doi: 10.1161/JAHA.120.019026 (PMC8649287; doi:10.1161/JAHA.120.019026)
Supplement: Supplementary file 1 — Data S1 Tables S1–S2 Figures S1–S12 [file JAH3-10-e019026-s001.pdf]

SUPPLEMENTAL MATERIAL

Data S1.

Supplemental Methods

Usual variability SBP using mixed effects model

Given a dataset with N individuals and n_i SBP measurements from the ith individual, $i = 1, \ldots, N$, let Y_{ij}, $j = 1, \ldots, n_i$, be the jth measurement of individual i taken at measurement time t_{ij}.

Consider a standard linear mixed effects model

$$Y_{ij} = \beta^T X_{ij} + b_i^T Z_{ij} + \varepsilon_{ij}$$

where X_{ij} is a covariate vector for the fixed effects β and Z_{ij} is a covariate vector for the random effects b_i, assumed normally distributed $b_i \sim N(0, \Sigma_b)$. The residual errors ϵ_{ij} are assumed independent and normally distributed, $\epsilon_{ij} \sim N(0, \sigma^2)$. We can allow variability in the repeated measurements to differ between individuals by replacing the residual SD σ with an individual-specific residual SD σ_i and assuming that the σ_i are randomly distributed. We assume a log-normal distribution for the residual SD distribution, ensuring positivity of the SDs, $\sigma_i \sim \log N(\mu_\sigma, \tau_\sigma^2)$. The choice of log-normal distribution also allows a natural extension of the model to incorporate correlation between the usual level and the residual SD by assuming a multivariate normal distribution for the random effects and log residual SD

$$\varepsilon_{ij} \sim N(0, \sigma_i^2), \qquad \begin{pmatrix} b_i \\ \log \sigma_i \end{pmatrix} \sim N\left(\begin{pmatrix} 0 \\ \mu_\sigma \end{pmatrix}, \begin{pmatrix} \Sigma_b & \Sigma_{b\sigma} \\ \Sigma_{b\sigma}^T & \tau_\sigma^2 \end{pmatrix} \right)$$

where $\Sigma_{b\sigma}$ is a vector of covariances between the random effects and the random residual errors. The details of the models have been described elsewhere in the literature [12].
For this study, the model was

$$\begin{pmatrix} b_i \\ \log \sigma_i \end{pmatrix} \sim N\left(\begin{pmatrix} 0 \\ \mu_\sigma \end{pmatrix}, \begin{pmatrix} \tau_0^2 & \rho\tau_0\tau_\sigma \\ \rho\tau_0\tau_\sigma & \tau_\sigma^2 \end{pmatrix} \right)$$

For the Bayesian estimation, we used diffuse uniform prior distributions U[0, 100] for SDs, uniform U[−1, 1] prior distributions for correlation parameters, and diffuse normal prior distributions N(0, 100^2) for all other parameters. Priors were specified for the bivariate and trivariate normal distributions by expressing them as two and three conditional univariate normal distributions, respectively. In the current study, we used a burn-in of 1000 Markov Chain Monte Carlo updates for the mixed effects models. The posterior means (95% credible interval) for usual SBP and SBP variability were 137.1 (112.9, 160.3) and 12.7 (7.1, 20.9), respectively.

Rosner's regression method

Rosner's regression method is used to calculate the regression dilution ratio to evaluate the association between systolic blood pressure (SBP) variability and event outcomes in sensitivity analysis 1. The detailed method is shown as below.

First, the standard deviation (SD) of SBP as the variability measurement was calculated based on the measurements of SBP at 9 visits (baseline, every 3 months in the past). To deal with random errors in this SBP variability measurement, regression dilution ratio was applied to the analyses based on Rosner's regression method [34], employing the measurements of SBP at 8 visits unit 2 years after baseline (every 3 months after baseline). The timeline for the measurement of SBP and outcome ascertainment of this method shown in the Figure below. Regression dilution ratio was calculated as the coefficient relating to the post measurement to the baseline measurement. Finally, continuous hazard ratios for baseline measurement were multiplied by the ratio to estimate the association for SBP variability. For example, regression dilution ratio of 4 for SD of SBP. If an hazard ratio for baseline SD of SBP of 1.1 was calculated, the hazard ratio for SBP was calculated as $e^{(4 \times \ln(1.1))} = 1.5$.

Figure. Study design for sensitivity analysis 1.

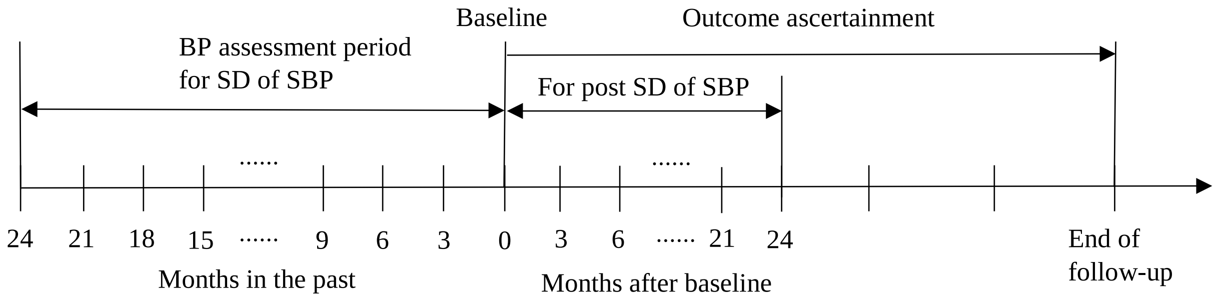

In current study, the mean of SD of SBP at post SD of SBP was 12.1mmHg (SD: 4.8mmHg). Applying the Rosner's regression method, the regression dilution ratio was 4.13. To evaluate the association between SBP variability and event outcomes, multivariable Cox proportional hazards regressions adjusted with baseline characteristics, usual SBP and regression dilution ratio. The results were shown in **Figure S5**.

Table S1. Definition of the diseases.

Event	ICPC-2 codes	ICD-9-CM codes
DM	T89, T90	NA
CHD	K74-K76	410-414
Heart Failure	K77	428
Stroke	K89-K91	430-438

ICPC-2 = the International Classification of Primary Care-2; ICD-9-CM = the International Classification of Diseases, Ninth Edition, Clinical Modification; HT = Hypertension; DM = Diabetes Mellitus; CHD = Coronary heart disease

Table S2. Data completion rate of the baseline characteristics in studied patients.

	Total (N = 155,982)
Age	100.0% (155,982)
Sex	100.0% (155,982)
Smoking status	97.4% (151,991)
BMI	64.6% (100,753)
SBP	100.0% (155,982)
DBP	100.0% (155,982)
HbA1c	84.3% (131,493)
LDL-C	63.6% (99,236)
eGFR	77.1% (120,190)
Charlson Index	100.0% (155,982)
Use of oral anti-diabetic drugs	100.0% (155,982)
Use of insulin	100.0% (155,982)
Use of ACEI/ARB	100.0% (155,982)
Use of β-blocker	100.0% (155,982)
Use of CCB	100.0% (155,982)
Use of Diuretic	100.0% (155,982)
Use of other anti-hypertensive drugs	100.0% (155,982)
Use of lipid-lowering agents	100.0% (155,982)

Other anti-hypertensive drugs included Acarbose, Glucagon-like peptide-1 agonist, Meglitinides. BMI = Body Mass Index; SBP = Systolic Blood Pressure; DBP = Diastolic Blood Pressure; HbA1c = Haemoglobin A1c; LDL-C = Low-density Lipoprotein-Cholesterol; eGFR = Estimated Glomerular Filtration Rate; ACEI = Angiotensin Converting Enzyme Inhibitor; ARB = Angiotensin Receptor Blocker; CCB = Calcium Channel Blocker

Figure S1. Study design for the investigation of the association visit-to-visit in systolic blood pressure (SBP) and cardiovascular diseases and all-cause mortality. The measurements of SBP at 9 visits (baseline, each 3 months in the past) were used to calculate usual mean and variability of SBP. The median follow-up period was 9.7 years after baseline

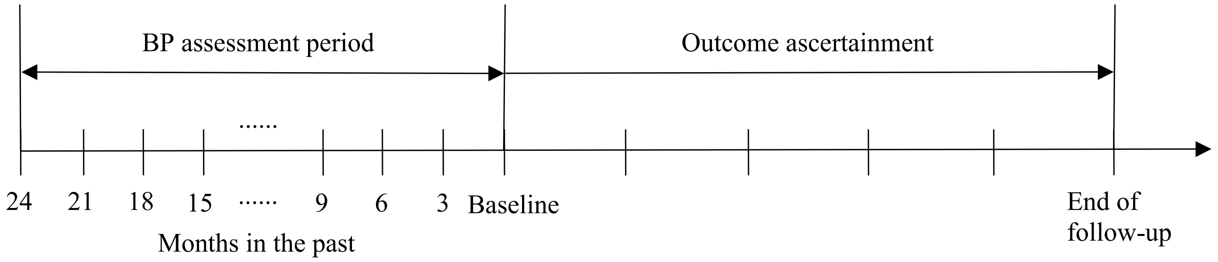

Figure S2. Adjusted Hazard ratio for incidence of CVD, CHD, Stroke, Heart failure, all−cause mortality, CVD mortality, non−CVD mortality and their composite with increasing usual SBP variability by multivariable Cox regressions.

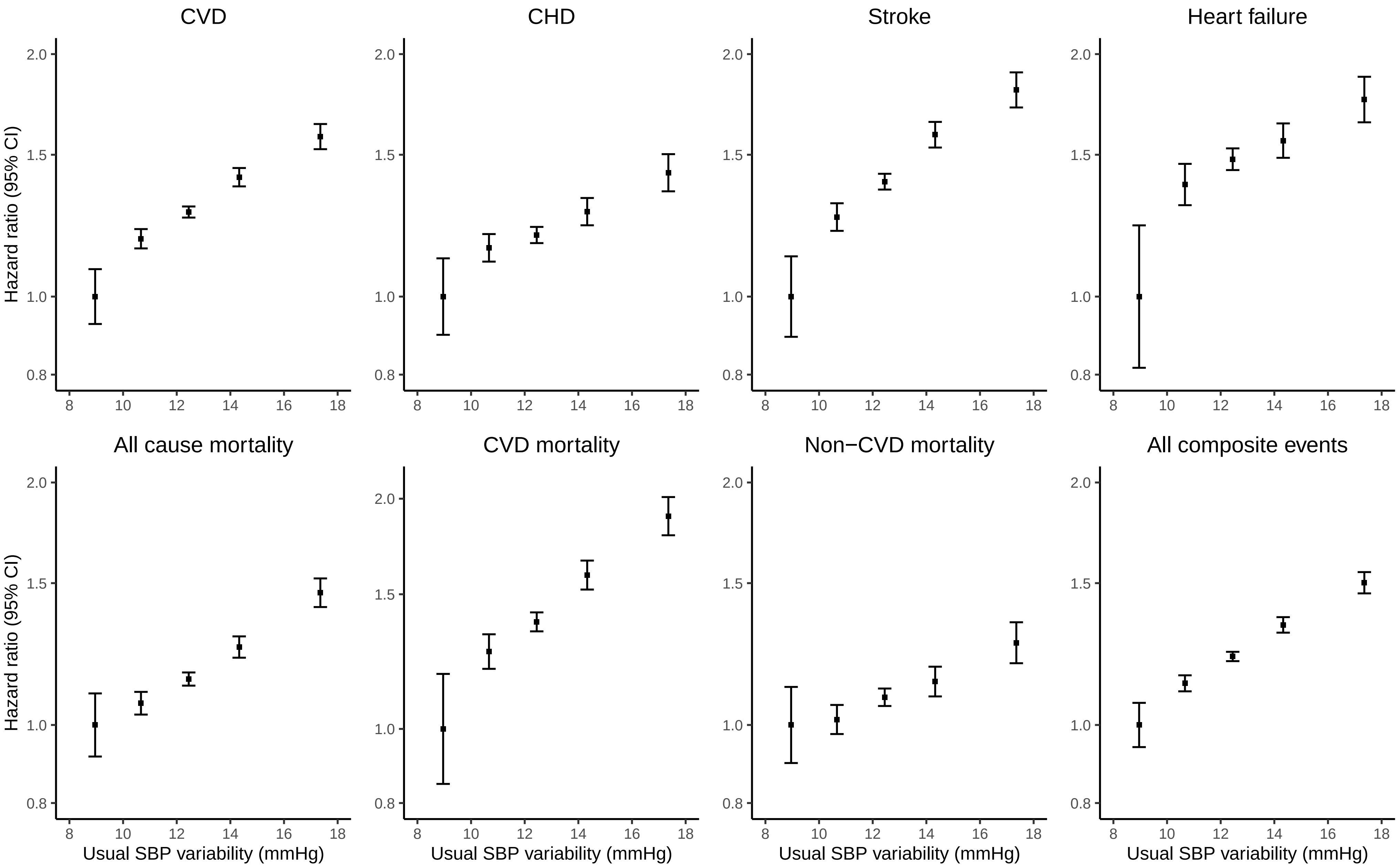

Hazard ratio was adjusted by age at risk, gender, smoking status, body mass index, SBP, diastolic blood pressure, haemoglobin A1c, low−density lipoprotein−cholesterol, estimated glomerular filtration rate, the usages of oral anti−diabetic drugs, insulin, angiotensin converting enzyme inhibitor/angiotensin receptor blocker, ß−blocker, calcium channel blocker, diuretic, other anti−hypertensive drugs, lipid−lowering agent, Charlson's index and usual SBP. CIs are displayed as floating absolute risks. SD=Standard deviation; SBP=Systolic blood pressure; CVD=Cardiovascular disease; CHD=Coronary heart disease.

Figure S3. Adjusted Hazard ratio for incidence of CVD, CHD, Stroke, Heart failure, all-cause mortality, CVD mortality, non-CVD mortality and their composite with increasing usual SBP variability by multivariable Cox regressions with restricted cubic spline.

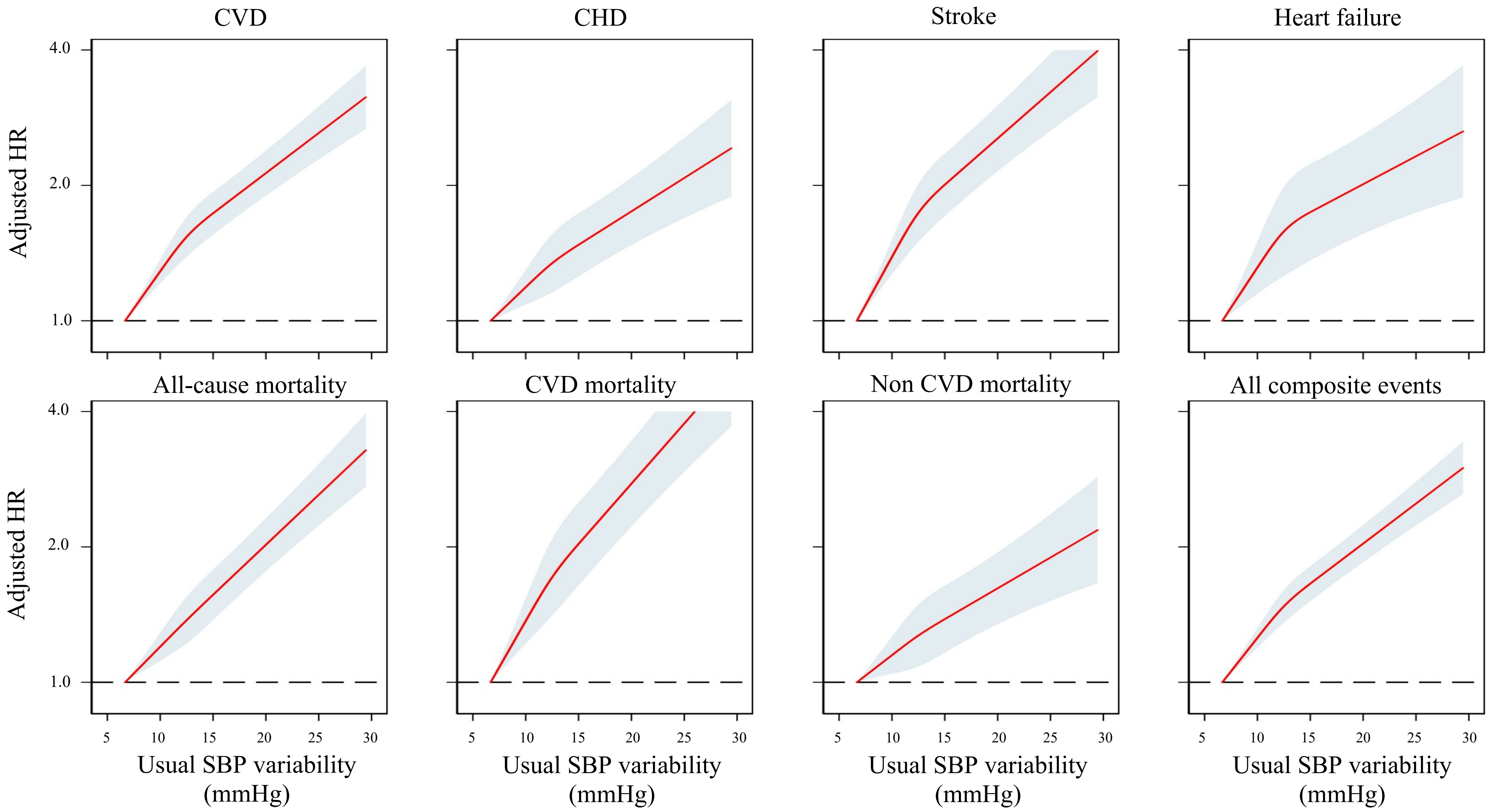

Hazard ratio was adjusted by age at risk, sex, smoking status, body mass index, SBP, diastolic blood pressure, haemoglobin A1c, low-density lipoprotein-cholesterol, estimated glomerular filtration rate, the usages of oral anti-diabetic drugs, insulin, angiotensin converting enzyme inhibitor/angiotensin receptor blocker, β-blocker, calcium channel blocker, diuretic, other anti-hypertensive drugs, lipid-lowering agent, Charlson's index and usual SBP. Shaded region represents 95% confidence intervals. SBP=Systolic blood pressure; CVD=Cardiovascular disease; CHD=Coronary heart disease;

Figure S4. Age-specific adjusted hazard ratios for the risk of CVD, coronary heart disease, stroke, heart failure, CVD mortality and non-CVD mortality with increasing usual SBP variability by multivariable Cox re-gressions.

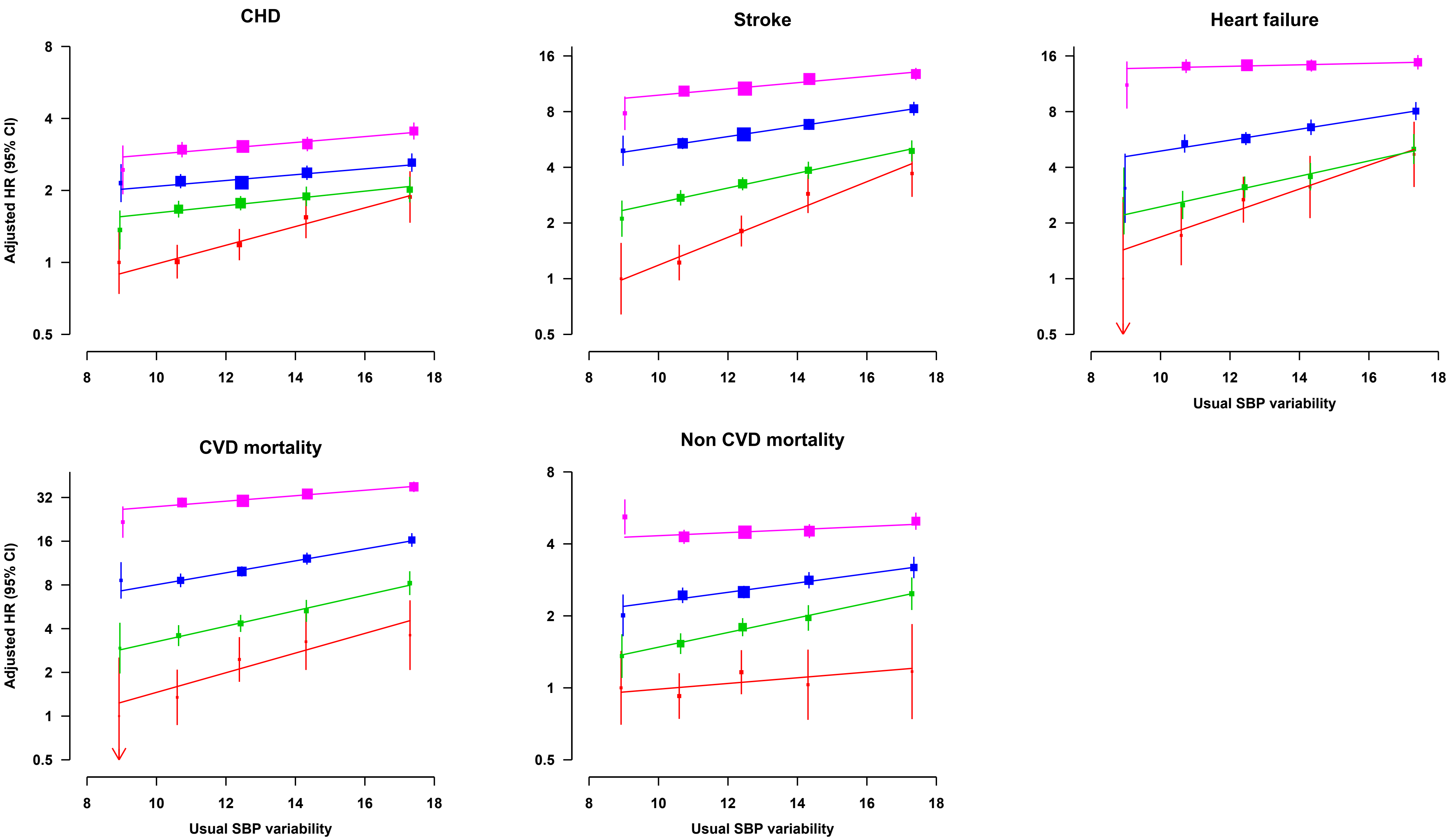

HR was adjusted by age at risk, sex, smoking status, body mass index, SBP, diastolic blood pressure, haemoglobin A1c, low-density lipoprotein-cholesterol, estimated glomerular filtration rate, the usages of oral anti-diabetic drugs, insulin, angiotensin converting enzyme inhibitor/angiotensin receptor blocker, β-blocker, calcium channel blocker, diuretic, other anti-hypertensive drugs, lipid-lowering agent, Charlson's index and usual SBP. Red, green, blue and pink line were 45-54, 55-64, 65-74 and 75-84 age at risk group. The area of each square was inversely proportional to the variance of the category-specific log risk. CIs are displayed as floating absolute risks. SBP=Systolic blood pressure; CVD=Cardiovascular disease; HR=Hazard ratio; CI=Confidence interval.

Figure S5. Adjusted hazard ratios for the risk of CVD, coronary heart disease, stroke, heart failure, all cause mortality, CVD mortality, non-CVD mortality and their composite with each 5mmHg increasing usual SBP variability using multivariable Cox regressions based on Rosner's regression method (Sensitivity analysis 1).

Outcome	Event	HR (95% CI)
CVD	34,039	1.33 (1.28, 1.38)
Coronary heart disease	15,897	1.26 (1.19, 1.33)
Stroke	17,152	1.40 (1.32, 1.47)
Heart failure	9,772	1.27 (1.18, 1.36)
All cause mortality	29,211	1.33 (1.27, 1.39)
Cardiovasular mortality	14,007	1.46 (1.37, 1.55)
Non-cardiovasular mortality	15,204	1.21 (1.14, 1.29)
**All composite events**	**49,816**	**1.31 (1.27, 1.35)**

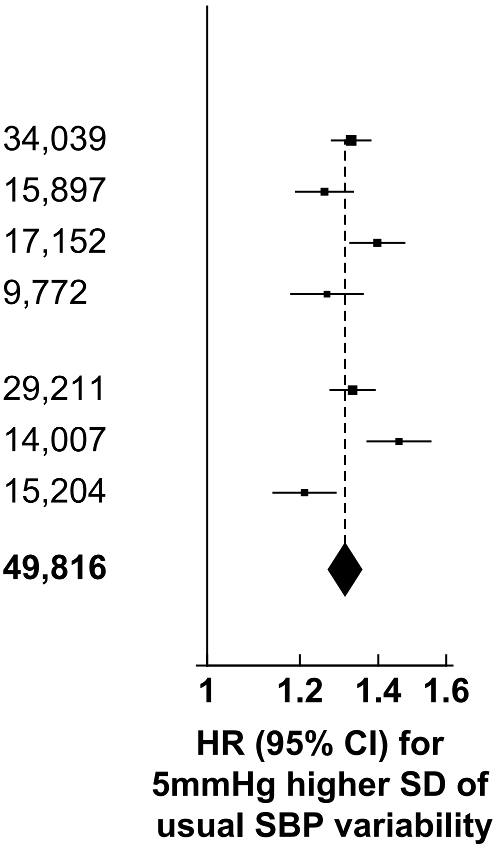

Applying the Rosner's regression method, the regression dilution ratio was 4.13.

HR was adjusted by age at risk, gender, smoking status, body mass index, SBP, diastolic blood pressure, haemoglobin A1c, low-density lipoprotein-cholesterol, estimated glomerular filtration rate, the usages of oral anti-diabetic drugs, insulin, angiotensin converting enzyme inhibitor/angiotensin receptor blocker, β-blocker, calcium channel blocker, diuretic, other anti-hypertensive drugs, lipid-lowering agent, Charlson's index, usual SBP and regression dilution ratio. SD=Standard deviation; SBP=Systolic blood pressure; CVD=Cardiovascular disease; HR=Hazard ratio.

Figure S6. Adjusted hazard ratios for the risk of CVD, coronary heart disease, stroke, heart failure, all cause mortality, CVD mortality, non-CVD mortality and their composite with each 5mmHg increasing usual SBP variability using multivariable Cox regressions based on complete case analysis (Sensitivity analysis 2).

Outcome	Event	HR (95% CI)
CVD	15,472	1.29 (1.23, 1.35)
Coronary heart disease	7,468	1.22 (1.13, 1.31)
Stroke	7,620	1.38 (1.29, 1.47)
Heart failure	4,231	1.25 (1.14, 1.37)
All cause mortality	12,004	1.30 (1.22, 1.37)
Cardiovasular mortality	5,662	1.43 (1.32, 1.55)
Non-cardiovasular mortality	6,342	1.16 (1.07, 1.26)
**All composite events**	**22,079**	**1.27 (1.22, 1.32)**

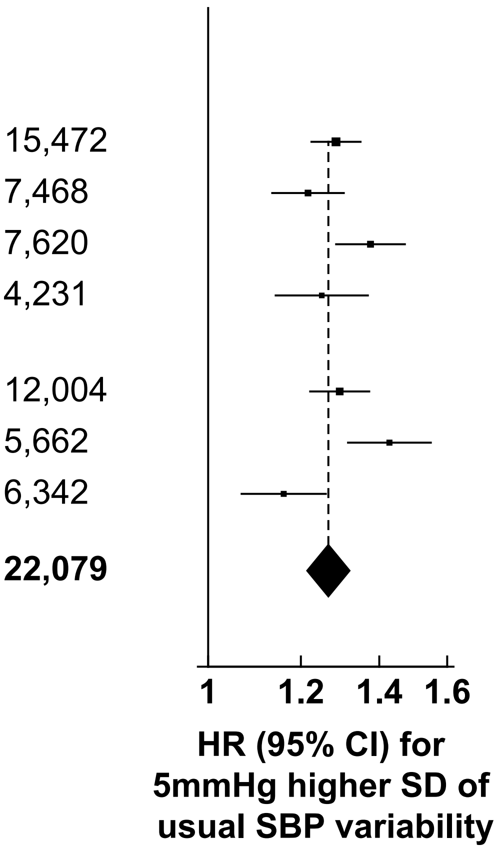

HR was adjusted by age at risk, sex, smoking status, body mass index, SBP, diastolic blood pressure, haemoglobin A1c, low-density lipoprotein-cholesterol, estimated glomerular filtration rate, the usages of oral anti-diabetic drugs, insulin, angiotensin converting enzyme inhibitor/angiotensin receptor blocker, β-blocker, calcium channel blocker, diuretic, other anti-hypertensive drugs, lipid-lowering agent, Charlson's index and usual SBP. SD=Standard deviation; SBP=Systolic blood pressure; CVD=Cardiovascular disease; HR=Hazard ratio.

Figure S7. Adjusted hazard ratios for the risk of CVD, coronary heart disease, stroke, heart failure, all cause mortality, CVD mortality, non-CVD mortality and their composite with each 5mmHg increasing usual SBP variability using multivariable Cox regressions based on patients with at least 12 months follow-up period (Sensitivity analysis 3).

**Outcome**	**Event**	HR (95% CI)
CVD	30,429	1.27 (1.22, 1.31)
Coronary heart disease	14,031	1.21 (1.15, 1.28)
Stroke	15,130	1.32 (1.26, 1.39)
Heart failure	8,759	1.18 (1.11, 1.26)
All cause mortality	26,289	1.27 (1.22, 1.33)
Cardiovasular mortality	12,181	1.39 (1.31, 1.48)
Non-cardiovasular mortality	14,108	1.16 (1.10, 1.23)
**All composite events**	**45,061**	**1.25 (1.21, 1.29)**

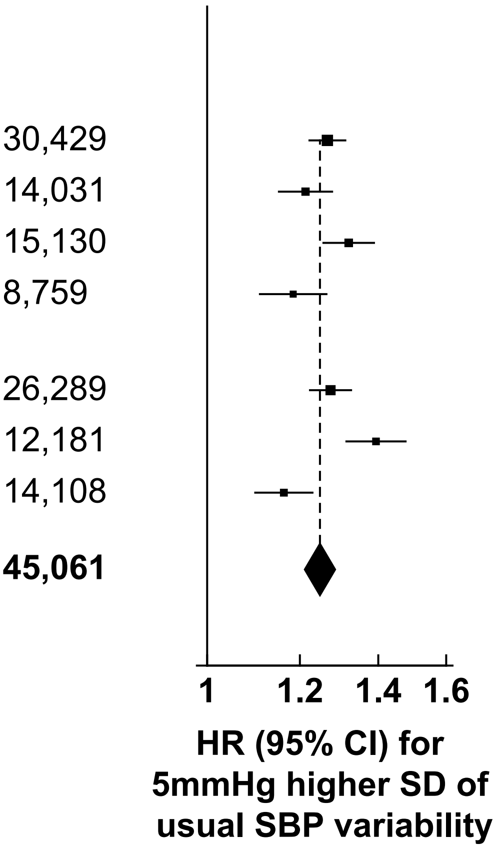

HR was adjusted by age at risk, sex, smoking status, body mass index, SBP, diastolic blood pressure, haemoglobin A1c, low-density lipoprotein-cholesterol, estimated glomerular filtration rate, the usages of oral anti-diabetic drugs, insulin, angiotensin converting enzyme inhibitor/angiotensin receptor blocker, β-blocker, calcium channel blocker, diuretic, other anti-hypertensive drugs, lipid-lowering agent, Charlson's index and usual SBP. SD=Standard deviation; SBP=Systolic blood pressure; CVD=Cardiovascular disease; HR=Hazard ratio.

Figure S8. Adjusted hazard ratios for the risk of CVD, coronary heart disease, stroke, heart failure, all cause mortality, CVD mortality, non-CVD mortality and their composite with each 5mmHg increasing usual SBP variability using multivariable Cox regressions based on patients with at least 2 SBP measurements on or before baseline (Sensitivity analysis 4).

Outcome	Event	HR (95% CI)
CVD	36,393	1.27 (1.23, 1.31)
Coronary heart disease	16,997	1.21 (1.16, 1.27)
Stroke	18,400	1.32 (1.27, 1.39)
Heart failure	10,409	1.20 (1.13, 1.27)
All cause mortality	31,211	1.31 (1.26, 1.36)
Cardiovasular mortality	14,974	1.40 (1.33, 1.48)
Non-cardiovasular mortality	16,237	1.20 (1.14, 1.27)
**All composite events**	**53,232**	**1.27 (1.23, 1.30)**

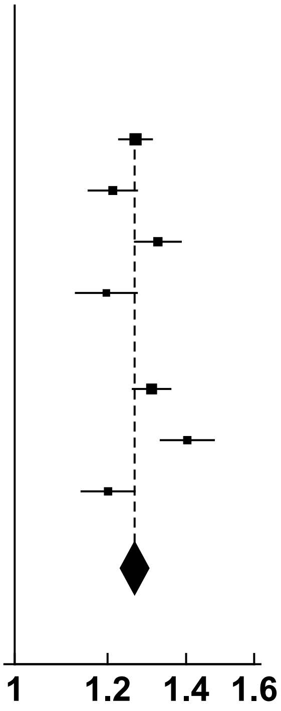

HR (95% CI) for 5mmHg higher SD of usual SBP variability

HR was adjusted by age at risk, sex, smoking status, body mass index, SBP, diastolic blood pressure, haemoglobin A1c, low-density lipoprotein-cholesterol, estimated glomerular filtration rate, the usages of oral anti-diabetic drugs, insulin, angiotensin converting enzyme inhibitor/angiotensin receptor blocker, β-blocker, calcium channel blocker, diuretic, other anti-hypertensive drugs, lipid-lowering agent, Charlson's index and usual SBP. SD=Standard deviation; SBP=Systolic blood pressure; CVD=Cardiovascular disease; HR=Hazard ratio.

Figure S9. Adjusted hazard ratios for the risk of CVD, coronary heart disease, stroke, heart failure, all cause mortality, CVD mortality, non-CVD mortality and their composite with each 5mmHg increasing usual SBP variability using multivariable Cox regressions based on patients with at least 7 SBP measurements on or before baseline (Sensitivity analysis 4).

Outcome	Event	HR (95% CI)
CVD	2,689	1.33 (1.21, 1.47)
Coronary heart disease	1,194	1.22 (1.05, 1.42)
Stroke	1,331	1.36 (1.18, 1.57)
Heart failure	721	1.44 (1.19, 1.74)
All cause mortality	1,949	1.19 (1.05, 1.36)
Cardiovasular mortality	816	1.33 (1.09, 1.62)
Non-cardiovasular mortality	1,133	1.10 (0.93, 1.30)
**All composite events**	**3,868**	**1.28 (1.18, 1.40)**

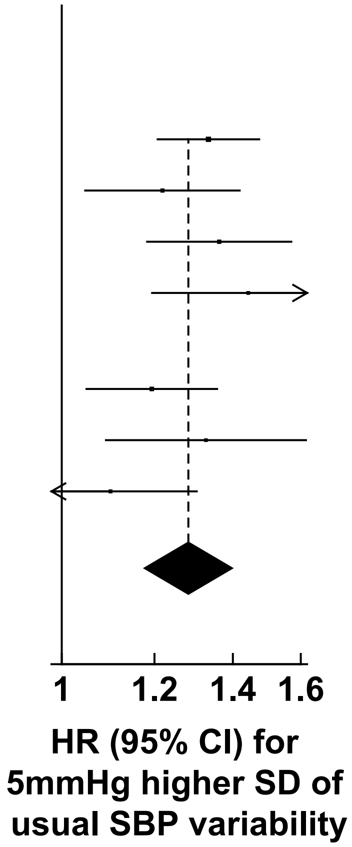

HR was adjusted by age at risk, sex, smoking status, body mass index, SBP, diastolic blood pressure, haemoglobin A1c, low-density lipoprotein-cholesterol, estimated glomerular filtration rate, the usages of oral anti-diabetic drugs, insulin, angiotensin converting enzyme inhibitor/angiotensin receptor blocker, β-blocker, calcium channel blocker, diuretic, other anti-hypertensive drugs, lipid-lowering agent, Charlson's index and usual SBP. SD=Standard deviation; SBP=Systolic blood pressure; CVD=Cardiovascular disease; HR=Hazard ratio.

Figure S10. Adjusted hazard ratios for the risk of CVD, coronary heart disease, stroke, heart failure, all cause mortality, CVD mortality, non-CVD mortality and their composite with each 5mmHg increasing usual SBP variability using multivariable Cox regressions with additional adjustment for patients with the usage of aspirin on or before baseline (Sensitivity analysis 5).

**Outcome**	**Event**	HR (95% CI)
CVD	34,039	1.25 (1.21, 1.30)
Coronary heart disease	15,897	1.19 (1.13, 1.25)
Stroke	17,152	1.32 (1.26, 1.38)
Heart failure	9772	1.20 (1.13, 1.27)
All cause mortality	29,211	1.29 (1.25, 1.34)
Cardiovasular mortality	14007	1.39 (1.31, 1.46)
Non-cardiovasular mortality	15,204	1.19 (1.13, 1.25)
**All composite events**	**49,816**	**1.25 (1.22, 1.29)**

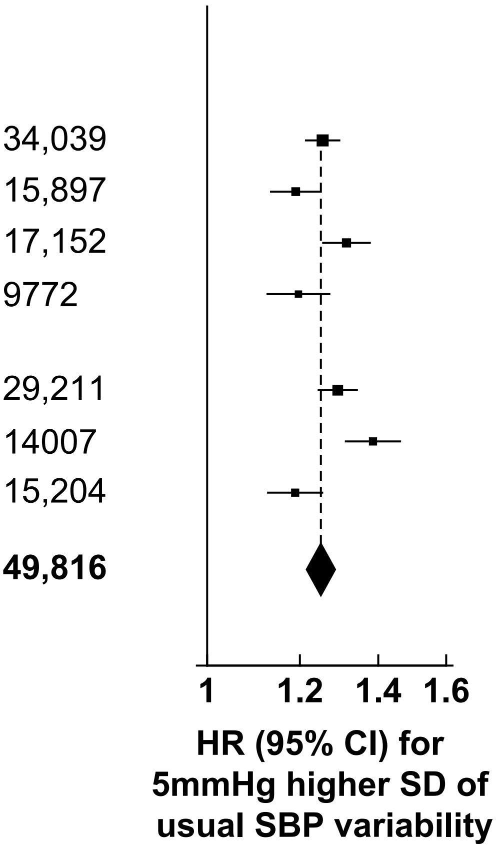

HR was adjusted by age at risk, sex, smoking status, body mass index, SBP, diastolic blood pressure, haemoglobin A1c, low-density lipoprotein-cholesterol, estimated glomerular filtration rate, the usages of oral anti-diabetic drugs, insulin, angiotensin converting enzyme inhibitor/angiotensin receptor blocker, β-blocker, calcium channel blocker, diuretic, other anti-hypertensive drugs, lipid-lowering agent, aspirin, Charlson's index and usual SBP. SD=Standard deviation; SBP=Systolic blood pressure; CVD=Cardiovascular disease; HR=Hazard ratio.

Figure S11. Adjusted hazard ratios for the risk of coronary heart disease, stroke and heart failure with each 5mmHg increasing usual SBP variability by stratifying patient's characteristics at baseline using multivariable Cox regressions.

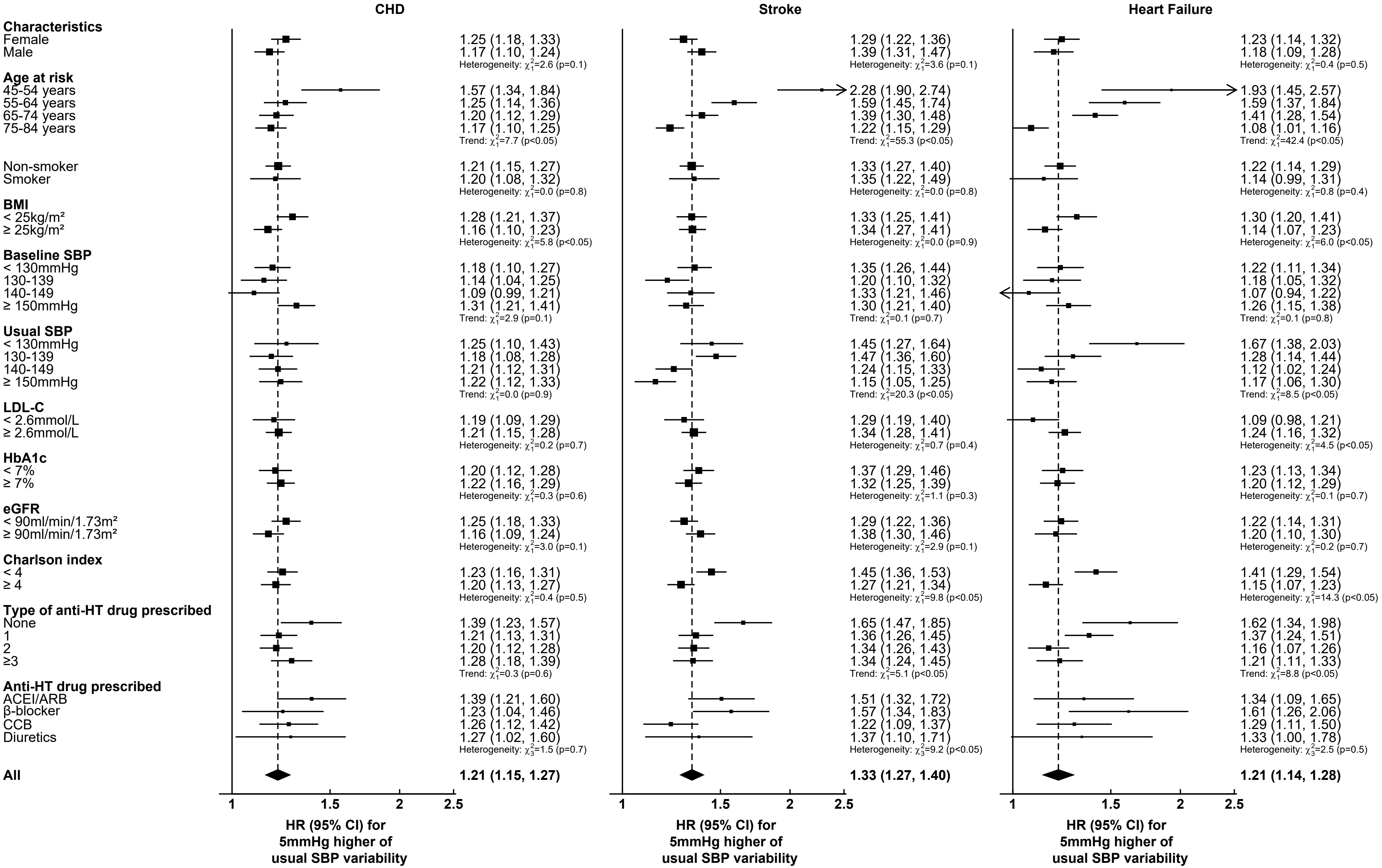

HR was adjusted by age at risk, sex, smoking status, BMI, SBP, diastolic blood pressure, HbA1c, LDL-C, estimated glomerular filtration rate, the usages of oral anti-diabetic drugs, insulin, angiotensin converting enzyme inhibitor/angiotensin receptor blocker, β-blocker, calcium channel blocker, diuretic, other anti-hypertensive drugs, lipid-lowering agent, Charlson's index and usual SBP. SBP=Systolic blood pressure; CHD=Coronary heart disease; BMI=Body Mass Index; LDL-C=Low-density lipoprotein-cholesterol; HbA1c=Haemoglobin A1c; HR=Hazard ratio; eGFR=estimated glomerular filtration rate; HT=Hypertensive; ACEI = Angiotensin Converting Enzyme Inhibitor; ARB = Angiotensin Receptor Blocker; CCB = Calcium Channel Blocker; CI=Confidence interval.

Figure S12. Adjusted hazard ratios for the risk of CVD mortality and non-CVD mortality with each 5mmHg increasing usual SBP variability by stratifying patient's characteristics at baseline using multivariable Cox regressions.

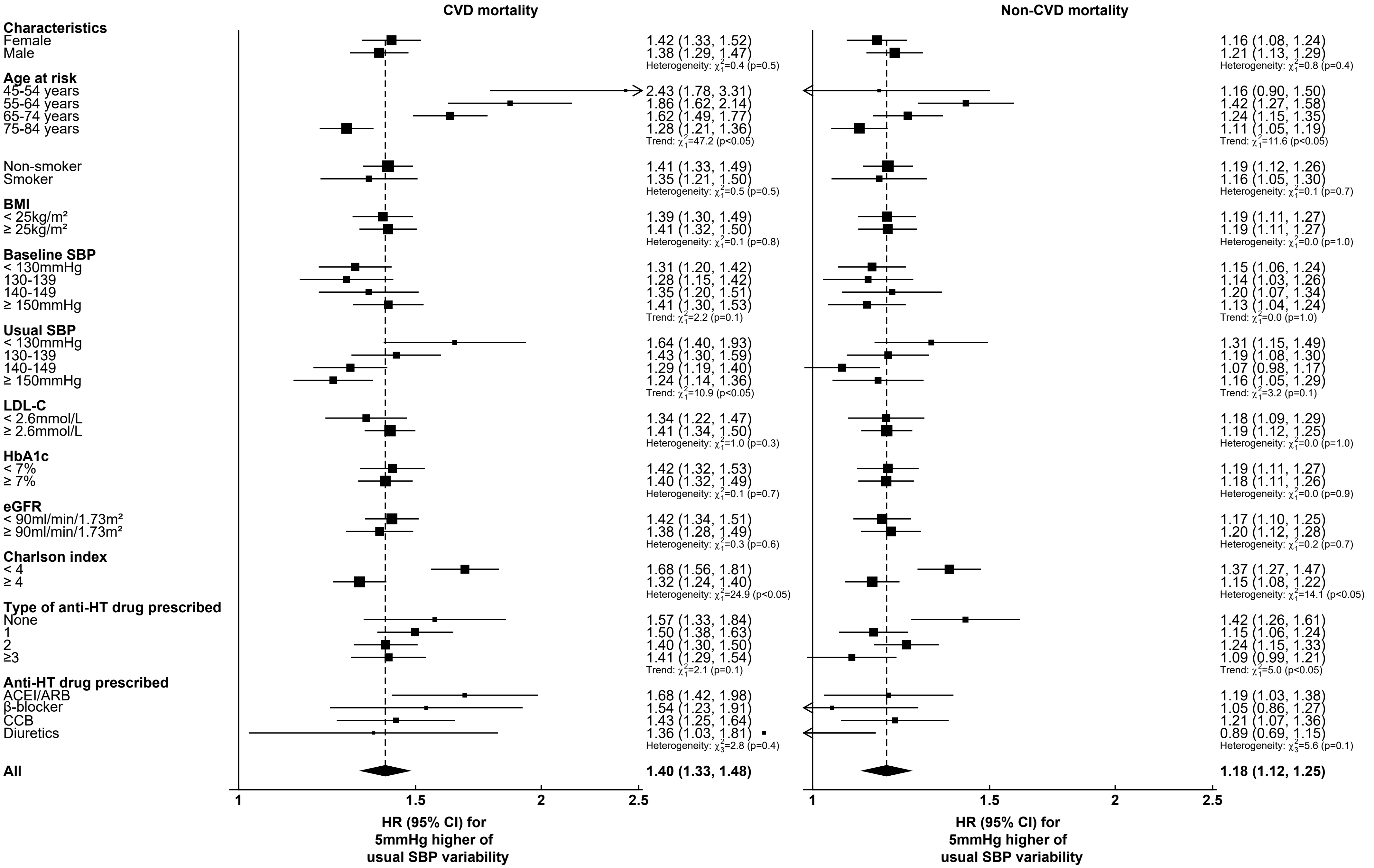

HR was adjusted by age at risk, sex, smoking status, BMI, SBP, diastolic blood pressure, HbA1c, LDL-C, estimated glomerular filtration rate, the usages of oral anti-diabetic drugs, insulin, angiotensin converting enzyme inhibitor/angiotensin receptor blocker, β-blocker, calcium channel blocker, diuretic, other anti-hypertensive drugs, lipid-lowering agent, Charlson's index and usual SBP. SBP=Systolic blood pressure; CVD=Cardiovascular disease; BMI=Body Mass Index; LDL-C=Low-density lipoprotein-cholesterol; HbA1c=Haemoglobin A1c; HR=Hazard ratio; eGFR=estimated glomerular filtration rate; HT=Hypertensive; ACEI = Angiotensin Converting Enzyme Inhibitor; ARB = Angiotensin Receptor Blocker; CCB = Calcium Channel Blocker; CI=Confidence interval.
